# Molecular Aspects of Circadian Pharmacology and Relevance for Cancer Chronotherapy

**DOI:** 10.3390/ijms18102168

**Published:** 2017-10-17

**Authors:** Narin Ozturk, Dilek Ozturk, Ibrahim Halil Kavakli, Alper Okyar

**Affiliations:** 1 Department of Pharmacology, Faculty of Pharmacy, Istanbul University, TR-34116 Beyazit-Istanbul, Turkey; narin.ozturk@istanbul.edu.tr (N.O.); dozturk@bezmialem.edu.tr (D.O.); 2 Department of Pharmacology, Faculty of Pharmacy, Bezmialem Vakif University, TR-34093 Fatih-Istanbul, Turkey; 3 Departments of Molecular Biology and Genetics and Chemical and Biological Engineering, Koc University, TR-34450 Sariyer-Istanbul, Turkey; hkavakli@ku.edu.tr

**Keywords:** biological rhythms, circadian timing system (CTS), chronopharmacodynamics, chronopharmacokinetics, cancer chronotherapy

## Abstract

The circadian timing system (CTS) controls various biological functions in mammals including xenobiotic metabolism and detoxification, immune functions, cell cycle events, apoptosis and angiogenesis. Although the importance of the CTS is well known in the pharmacology of drugs, it is less appreciated at the clinical level. Genome-wide studies highlighted that the majority of drug target genes are controlled by CTS. This suggests that chronotherapeutic approaches should be taken for many drugs to enhance their effectiveness. Currently chronotherapeutic approaches are successfully applied in the treatment of different types of cancers. The chronotherapy approach has improved the tolerability and antitumor efficacy of anticancer drugs both in experimental animals and in cancer patients. Thus, chronobiological studies have been of importance in determining the most appropriate time of administration of anticancer agents to minimize their side effects or toxicity and enhance treatment efficacy, so as to optimize the therapeutic ratio. This review focuses on the underlying mechanisms of the circadian pharmacology i.e., chronopharmacokinetics and chronopharmacodynamics of anticancer agents with the molecular aspects, and provides an overview of chronotherapy in cancer and some of the recent advances in the development of chronopharmaceutics.

## 1. Introduction

Circadian rhythms are oscillations in the behavior and biochemical reactions of organisms, and occur with a periodicity of approximately twenty-four hours (cf Latin circa, or “about”, and dies, or “day”) [1,2]. Three important properties of the circadian rhythm are: (i) its innate nature, that is, the intrinsic rhythm that exists without any sensory input from the environment; (ii) its capacity for temperature compensation, that is, the maintenance of the intrinsic period, phase, and amplitude of the rhythm despite external fluctuations in temperature, so long as these fluctuations do not interfere with physiological thermoregulation; and (iii) its photoentrainment, that is, the synchronization of the phases of the rhythm with the external light-dark cycles of the solar day [3,4,5,6]. Studies in the field of biological rhythms over the last decade have shown that many aspects of human biology have a circadian rhythm. We wake up, we are active, and we sleep at very similar times each day. Because the environmental changes that accompany the day are so predictable, nature has devised an ingenious system, the circadian clock, to anticipate these changes and regulate physiology and behavior accordingly [3,4,5]. For example, before we wake up, our heart rate and blood pressure increase in anticipation of this stressful event [6]. Likewise, our mental state and capacity for memory and learning, as well as our state of arousal and onset of sleep, are also clock-regulated [7,8,9]. The circadian clock regulates the timing of sleep and wakefulness, and therefore all dependent behavioral and physiological processes. In humans, a defect in the clock gene Period (*PER2*) produces familial advanced sleep phase syndrome (FASPS) [10,11], and an analogous mutation causes the same phenotype in mice [12]. People with a causal mutation in casein kinase, *CSNK1D*, and an associated variant, *CSNK1E*, display advanced sleep phase syndrome (ASPS) and delayed sleep phase syndrome (DSPS), respectively [13,14]. Finally, a human Circadian Locomotor Output Cycles Kaput (*CLOCK*) variant is associated with diurnal sleep preference [15]. Circadian clock genes are also associated with a host of neurological disorders including schizophrenia [16,17,18,19], unipolar major depression [20], and bipolar disorder [21,22]. Abnormalities in the circadian rhythms are also closely related with the pathophysiology of cancer [23,24,25]. Impairment of the CTS in cancer patients is concerned with many various systemic symptoms with the inclusion of fatigue, sleep disorders, body weight loss due to appetite loss in addition to poor therapeutic outcomes [23,26,27,28].

The toxicity of anticancer agents in healthy cells and the efficacy in malignant tumors may be modified depending on drug administration time in both experimental models and in cancer patients [27,28,29,30,31,32]. When drugs are given at their respective times of best tolerability, improved efficacy is seen [29]. Circadian rhythms of biological functions may affect pharmacokinetics, target organ toxicity and antitumor activity of anticancer agents as a function of dosing time [29,33,34,35]. Several physiological and molecular mechanisms involved in the regulations of pharmacological processes under the control of circadian clock, beginning with the entry of the anticancer drug to the organism and ending with the clinical response and/or toxic effects, have started to emerge only recently [29,32,35,36,37,38]. Today, several chronotherapeutic approaches are successfully applied in the treatment of many cancers [27,32,36,38]. Chronotherapeutics consists in the delivery of medications as a function of circadian rhythms in order to minimize adverse effects and/or to enhance efficacy. Randomised clinical trials revealed that chrono-modulated delivery of anticancer chemotherapy protocol was 5-fold less toxic and nearly twice as effective as compared with constant rate infusion in male patients [39,40]. This review focuses on the underlying mechanisms of the circadian pharmacology of anticancer agents and provides an overview of chronotherapy in cancer and some of the recent advances in the development of chronopharmaceutics.

## 2. The Circadian Timing System (CTS) and Molecular Mechanisms of Circadian Clock

**Figure 1:**
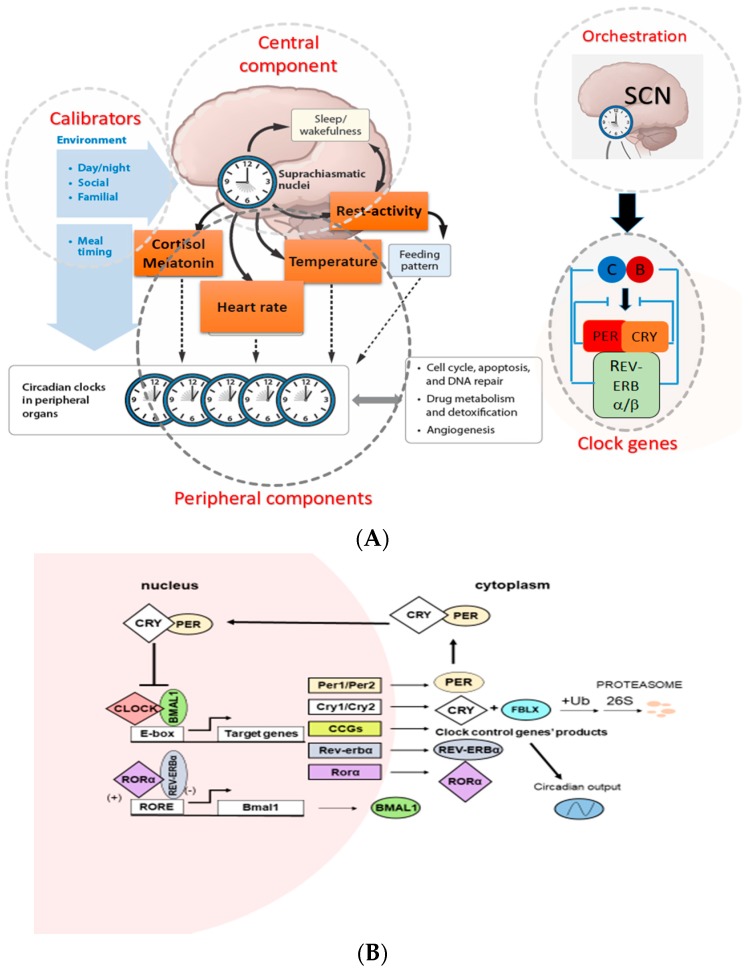
Hierarchical organization of the CTS and its calibrators in mammals. (**A**) The CTS consists of three components: an input component, a clockwork and output component. The input component mediated at the macroscopic level by the visual perception of light. Additionally, timing of the meal and social interaction are considered as the input, which they act as Zeitgeber. The second one, the master circadian clock is located in the hypothalamus as a pair of neuron clusters known as the suprachiasmatic nuclei (SCN). The circadian clock in SCN is entrained at precisely 24-h by environmental factors. The output component where SCN synchronizes and coordinates the molecular clocks in the peripheral tissues with a complex interplay between behavioral, neuroendocrine and neuronal pathways as well as SCN-generated physiological rhythms such as body temperature and rest-activity rhythms, heart rate, cortisol and melatonin secretion called as circadian biomarkers. Molecular clocks rhythmically control xenobiotic metabolism and detoxification, cell cycle, apoptosis, DNA repair, and angiogenesis over a 24-h period. Adapted from [30]. (**B**) Circadian oscillator is composed of interlocked positive and negative transcriptional feedback loops. In positive transcriptional feedback loops this CLOCK or NPAS4 and BMAL1 form a dimer and bind E-box of the clock controlled genes (CCGs) including *Per* and *Cry*. Then CRY and PER along with casein kinase ε (CKIε) form a trimeric complex and moves into nucleus, where it suppresses the activity of BMAL:CLOCK activity, which forms negative transcriptional feedback loop. A second feedback loop is formed by the action of orphan receptors, ROR*α* and REV-ERBα proteins, on *Bmal1* transcription. RORα and REV-ERB activate and repress transcription of *Bmal1* through their competitive action on response elements (ROREs) on the *Bmal1* promoter, respectively. Adapted from [52].

In mammals, the CTS are organized hierarchically, with light-responsive “master” pacemaker clocks located within the suprachiasmatic nucleus (SCN) of the hypothalamus that drive rhythmic cycles within extra-SCN neurons and peripheral tissues. The SCN aligns peripheral tissue clocks with the environmental light cycle through a combination of direct autonomic nervous system efferents and neuroendocrine signals (Figure 1A). At the molecular level, the clockwork of the cell involves several proteins that participate in positive and negative transcriptional feedback loops (Figure 1B). Brain and muscle aryl hydrocarbon receptor nuclear translocator 1 (BMAL1) and CLOCK are transcription factors that contain two basic helix-loop-helix domains and bind E-box elements (CACGTG) in the Period (*Per*) and Cryptochrome (*Cry*) genes and thereby exert a positive effect on circadian transcription [41,42,43]. The mammalian PER and CRY proteins act as negative regulators of transcription driven by the BMAL1/CLOCK heterodimer [44,45,46]. PER and CRY form heterodimers that interact with casein kinase Iε (CKIε) and then translocate into nucleus where CRY acts as a negative regulator of BMAL1/CLOCK–driven transcription [47]. In addition to the primary biochemical feedback loop that regulates cycling at the E-box, circadian gene expression is mediated by the transcription at the ROR/REV-ERB and the DBP-E4BP4 (D-box) binding elements NR1Ds and RORs (subfamilies of nuclear hormone receptors) which either activate or repress gene transcription form the ROR components in several clock genes [48]. Microarray studies have addressed the role of specific clock genes in peripheral oscillator function. For example, oscillations were lost in 90% of transcripts when the circadian clock was selectively disrupted in mouse hepatocytes, suggesting that the remaining 10% of rhythms were driven by systemic cues originating outside the liver but impinging on its function [49]. Genetic and bioinformatics studies suggested that there are more clock genes in mammals, which directly regulate core clock mechanisms or posttranslationally regulate core clock protein activity [50,51].

## 3. Experimental Chronopharmacology of Anticancer Drugs

**Figure 2:**
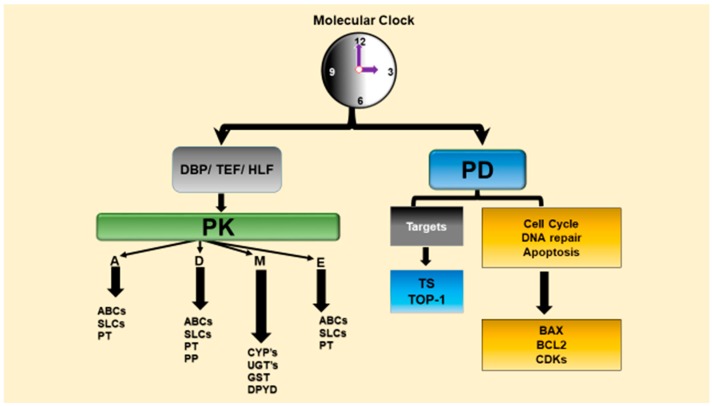
Molecular clock controls PK and PD of anticancer agents. The clock controls main pathways responsible for anticancer drug PK during the 24 h. Circadian clocks also controls several drug targets including cell cycle, DNA repair and apoptosis genes which are contributed to anticancer drugs’ PD. ABC: ATP-Binding Cassette, SLC: Solute Carriers, CYP: Cytochrome, UGT: Uridine diphosphate (UDP) glucronyl transferase, GST: Glutation S-transferase, DPYD: Dihydropyrimidine dehydrogenase, PT: Passive Transport, PP: plasma protein binding, TOP-1: topoisomerase 1, TS: thymidylate synthase, CDK: Cyclin-dependent kinase.

Chronopharmacology is a branch of pharmacology that deal with the biological rhythm dependencies of drugs and examines circadian variations of drug PK (chronoPK), drug PD (chronoPD) and toxicity (chronotoxicity) [1,29,53,54]. The CTS determines the optimal dosing times of several drugs including anticancer drugs via controlling drug metabolism and detoxification, drug transport, bioactivation, elimination, and molecular drug targets that account for the chronopharmacology (chronoPK and chronoPD) (Figure 2) [29,32,35,53]. We review the in vitro and in vivo studies and present possible molecular mechanisms behind the circadian time-dependent PK and PD of anticancer drugs.

The circadian physiology and the genes involved in the metabolism (e.g., CYPs), transport (e.g., ABC carriers) and elimination of the anticancer drugs, as well as those that are involved in the cell cycle events and apoptosis and other molecular drug targets (e.g., thymidylate synthase for fluoropyrimidines) are controlled by the CTS [23,34,35,55,56,57]. Indeed, diurnal oscillations in drug absorption, distribution, metabolism, and excretion (ADME), as well as daily variations in the sensitivity of molecular drug targets lead to the dosing-time dependent changes in the anticancer drug efficacy and safety [32,34,35,58].

The cell cycle events and apoptosis process in bone marrow and tumor such as WEE1, cyclin-dependent kinase 2 (CDC2), P21, BCL-2, BCL-2-associated X protein (BAX) and/or other molecular drug targets that are controlled by the molecular circadian clocks (Figure 2), and therefore transcriptions of these genes show circadian variations [23,29,32,34,55,56,57]. In fact, a recent genomics studies revealed that 56 of the top 100 best-selling drugs are the target product of a circadian gene in the United States. Moreover, they also showed that the half-lives ≈ 50% of these drugs are less than 6 h [59]. These suggest that PK and PD of these drugs with short half-life should be evaluated according to CTS to find their most effective dosing time. Short elimination half-life drugs will reach the protein in the correct time i.e., the highest expression level of protein, and shortly interact with them. Diurnal oscillations in drug absorption, distribution, metabolism, and excretion, as well as daily variations in the sensitivity of molecular drug targets lead to the dosing-time dependent changes in the drug effectiveness and safety [29,32,58].

### 3.1. Interaction of Circadian Clock Network with Drug Metabolism, Detoxification and Transport

The CTS modifies ADME of drugs over a 24-h period, which provides the molecular basis for dosing time-dependency of drug toxicity and efficacy [29,32,35]. Biological rhythms in drug metabolism, detoxification and drug transport manifest itself in circadian PK and PD of drugs, hence produce circadian changes in drug effectiveness and toxicity [28]. In mammals, biotransformation/detoxification processes involve chemical changes of xenobiotics/drugs in various organs such as liver, intestine, and kidney. The main results of such metabolism are the increase in xenobiotics/drugs’ water solubility and the facilitation of their excretion into bile, feces and/or urine by the transporters [53]. Phase I metabolism reactions involve enzymes from 27 different genetic families, of which 150 members are responsible for oxidation, reduction and hydrolysis (Class I). In particular, cytochrome P-450 (CYP450) microsomal enzyme superfamily plays a crucial role through its high expression in liver and intestine. Phase II metabolizing or conjugating enzymes such as UDP-glucuronosyl transferases (UGTs), glutathione-S-transferases (GST), sulfotransferases (SULT), and N-acetyl transferases (NAT) are responsible for conjugation (Class II). Phase III components consist of ATP-Binding Cassette (ABC) transporters and solute carrier (SLC) super family which are responsible for cellular efflux and influx of xenobiotics/drugs, respectively (Class III) [29,35,60,61]. In addition, P450 oxidoreductase (POR) and aminolevulinic acid synthase (ALAS1) enzymes take role in the regulation of the activity of most of the Phase I enzymes [35].

Circadian expression of the Phase I, II, and III detoxification proteins are regulated by DBP, HLF, and TEF proteins which are the three members of the PARbZip transcription factor family [62,63,64,65]. PARbZip transcription factors regulate the transcription of numerous key genes involving in the Phase I and Phase II drug metabolism, and drug transport by rhythmically binding to D-box-containing promoters of these genes [64]. Gachon et al. [66] showed that PARbZip-triple knockout mice were highly vulnerable to harmful effects of anticancer drugs. These knockout mice had highly reduced capacity to metabolize xenobiotics [66]. The molecular clocks in liver, kidney and small intestine which are the three pivotal tissue for xenobiotic detoxification, control the circadian expression of these PARbZip transcription factors. In turn, PARbZip proteins govern the circadian expression of three well-known nuclear receptors involved in the control of drug metabolism including constitutive androstan receptor (CAR), peroxisome proliferator activated receptor-alpha (PPAR-α), and aryl hydrocarbon receptor (Ahr) [62,64,65,66,67]. However, how the PARbZip proteins drive the rhythmic expression of pregnane X receptor (PXR) is not known precisely, i.e., mechanisms facilitating rhythmic transcription of PXR targets generally are not well-understood. A recent study by Kriebs et al. [68] revealed that CRY1 interacts with more than half of all mouse nuclear receptors directly, suggesting that direct repression of PXR by CRY1/2 enables circadian modulation of this nuclear receptor, and also pose an additional avenue for circadian clock regulation of rhythmically transcribed other nuclear receptors, including CAR. Gorbacheva et al. [69] indicated that mice with a *Bmal1* null allele or a mutation of the *Clock* gene exhibited enhanced sensibility to the toxic effect of the anticancer drug cyclophosphamide, however mice lacking of two *Cry* genes were more resistant to the toxic effect. These findings suggest that the circadian clock is directly involved in the chronotoxicity of drugs, via regulating genes involved in drug metabolism directly or through the circadian clock-regulated transcription factors [35]. PARbZip transcription factors also control the rhythmic expression of ALAS1 and POR in the mRNA and/or protein level [66]. 

Nuclear receptors function as xenobiotic sensors. In response to xenobiotic binding or activation signals induced by xenobiotics, they accumulate in the nucleus and activate the transcription of Phase I, II and III genes involving in the drug metabolism and transport [35,61,70]. The nuclear receptors REV-ERBα/β and RORα/γ are key regulators of core clock function, and many other nuclear receptors are rhythmically expressed under the control of core clock components [64,65,67]. In the RORα/γ double-knock out mice, major effects on gene regulation were observed as activation of *Cyp2b9/10*, *Cyp4a10* and *Cyp4a14*, *Sult1e1/Est* and *Sult2a9/2a*, and suppression of *Cyp7b1*, *Cyp8b1*, Hsd3b4 (3β-hydroxysteroid dehydrogenase) and Hsd3b5 [71]. Diurnal mRNA expression profiles of 49 nuclear receptors in the liver, skeletal muscle, white fat and brown fat tissues of mice were investigated in a detailed analysis with High-Throughput Real-Time RT-PCR (Reverse Transcriptase-Polymerase Chain Reaction). More than 50% of nuclear receptors analyzed in the study exhibited rhythmic variations with tissue-specific oscillation in these metabolically active tissues [72]. Two key nuclear receptors, CAR and PPAR-α are responsible for modulating rhythmic expressions of CYP450 and ABC transporters involving in the xenobiotic detoxification [29,65,67,73]. The temporal mRNA oscillation of the CAR is resemble to temporal changes of *Cyp1a* and *Cyp1b* mRNA expressions in rat liver [74]. Cyp2b10 activity is highest at night and lowest at daytime in the mouse liver, which is a direct target of CAR [66]. *Cyp2a5*, *Cyp2b10*, and *Cyp3a11* which show circadian rhythms in their expressions are the best-known targets of CAR nuclear receptor [75].

Transcriptome profiling elicited that PARbZip-deficient mice display a general down-regulation of genes coding enzymes involved in xenobiotic detoxification in the liver and kidney [66]. These genes include the Phase I (*Cyp2b*, *Cyp2c*, *Cyp2a* and *Cyp3a*), Phase II (*Ces3*, *GSTt1*, *and GSTa3*) and Phase III (*Abcg2*) group of detoxification components. The expression of *POR* and *ALAS1* is also decreased [66]. Recent findings suggested that PARbZip proteins directly regulate the expression of some of these enzymes by rhythmic binding to their promoter regions, for instance, in the case of *Cyp3a4* [76] and *Mdr1a* (multi-drug resistance 1a or Abcb1a) genes [67]. DBP activates the transcription of the human *Cyp3a4*, its mouse homolog *Cyp3a11*, and rat *Cyp2c6* genes through binding to their transcriptional start sites [77]. Lavery et al. [78] showed that DBP controls circadian regulation of *Cyp2a4* and *Cyp2a5* in mouse liver, where in Dbp null mice both genes showed significantly impaired expression. The mammalian CYP450 superfamily is mainly involved in xenobiotic metabolism, eicosanoid, cholesterol and bile acid biosynthesis, and arachidonic acid metabolism [77]. CYP450 is the major enzyme superfamily able to catalyze the oxidative biotransformation of most drugs, accounting for about ~75% of the total metabolism [79,80]. In human hepatoma (HepG2) cell line synchronized with serum shock, DBP activity and *CYP3A4* transcription were highest in daytime. Conversely, the CYP3A4 suppressor E4BP4 expression was highest at night [76]. Most of the anticancer drugs that undergo oxidation, reduction, or hydrolysis reactions under Phase I metabolism in the liver, and to some extent, in the intestine, show circadian tolerability [29]. Cyp3a enzyme shows the chronotolerance patterns of seliciclib, docetaxel, irinotecan, mitoxantrone, and vinorelbine by oxidative metabolism [29]. PARbZip-deficient mice are found to be extremely susceptible to anticancer drug-induced toxicity such as cyclophosphamide and mitoxantrone [66]. On the other hand, non-rhythmic *Cyp3a13*, rhythmic *Cyp2b10*, and possibly *Cyp2c29* participate in the circadian tolerability of cyclophosphamide [66]. The temporal microsomal oxidase and reductase expressions and activities of most of the CYP450 enzymes including *Cyp1a*, *Cyp2a*, *Cyp3a11*, *Cyp4a14*, *Cyp2b10* mRNA levels and activity of Cyp2e1 are higher during the dark (activity) span and lower during the light (rest) span in the liver and kidney of rats or mice [29]. In vitro and in vivo studies involving the circadian rhythms in mRNA expressions, protein levels and activities of CYP450 enzymes are reviewed in Table 1.

The transcriptional level of the major exsorptive ABC transporters and P-gp (P-glycoprotein, MDR1, ABCB1), which play an important role in the excretion of xenobiotics and expels drugs from blood to the intestinal lumen, respectively, are under the control of the CTS. P-gp is indirectly involved into xenobiotic metabolism resulting in the detoxification of potentially harmful substances by such “P-gp-CYP alliance”. Recent studies at the cellular level show that gene expression of ABC transporters including *abcb1a (mdr1a)*, *abcb1b (mdr1b)*, *abcc1* (Multidrug resistance-associated protein *1; mrp1)*, *abcc2 (mrp2)*, *abcb4 (mdr2)*, *bcrp1* are clock-controlled in mouse total ileum, ileal mucosa and liver [31,60,104], total jejunum of rats [105,106,107], and liver of monkeys [108]. *Abcb1a* transcription was shown to be rhythmic in mouse total ileum as a result of its alternative activation by HLF and repression by E4BP4. The role of clock on *abcb1a* circadian transcription was further demonstrated, since the *abcb1a* rhythm was disrupted in *Clock/Clock* mutant mice as compared to wild-type ones [67]. Ando et al. [109] found a clear 24 h rhythmicity in the *abcb1a* mRNA expression with a peak at ZT12-16 in the liver and intestine of C57BL/6J mice. Additionally, they also showed P-gp activity is circadian dependent where its substrate digoxin concentration was lower at ZT12. Studies involving the circadian rhythms in mRNA expressions, protein levels and activities of ABC transporters are summarized in Table 2.

SLC transporters are responsible for the influx of drugs in the intestine (absorption), cellular import of xenobiotics to hepatocytes, and reabsorption and excretion of xenobiotics in kidney. Import of xenobiotics to the liver possess particular importance since they metabolize in the hepatocytes. Influx of drugs via organic anion transporter polypeptide 1a2 (Oatp1a2) is important for liver metabolism of drugs. Zhang et al. [60] documented the daily mRNA expression rhythms of basolateral *Oatp1a1 (Slco1a1)*, *Oatp1a4 (Slco1a4)*, *Oatp1b2 (Slco1b2)*, *Oatp2b1 (Slco2b1)*, *Oat2*; *(Slc22a7)* and organic cation transporter-1 *(Oct1*; *Slc22a1)* in the mouse liver. Oct1 also display significant time dependent oscillation in the kidney, and clock rhythmically drives the renal expression of Oct1 protein through mediation PPARα. Likewise, daily intestinal mRNA expression of Oct novel type 1 (*Octn1*; *Slc22a4*) is governed by PPARα [110].

The carboxylesterases Ces1 and Ces2 are controlled rhythmically by CTS through PARbZip proteins in the liver and gastrointestinal tract [29,64]. Rhythmic expressions of *Ces1* and *Ces2* may be responsible for enhanced biotransformation of irinotecan into SN-38 during day time in male ICR mice [29,117]. Circadian detoxification of irinotecan was also observed in in vitro studies [112,113]. They showed in Caco-2 cells that the bioactivation (CES) and detoxification (UGT1A) enzymes of irinotecan were controlled by clock genes. Bioactivation of irinotecan by CES is maximum near the nadir of its detoxification enzyme UGT1A, which cause increased cytotoxicity. This led enhanced biotransformation to active metabolite SN38 and almost 4-fold change of irinotecan-induced apoptosis depending on the drug administration time [32]. Dihydropyrimidine dehydrogenase (DPYD) is another important circadian-controlled Phase I enzyme, responsible for detoxification of 5-fluorouracil (5-FU) and capecitabine [32]. Its transcription and activity are controlled by CTS in the liver of male *B6D2F1* mice. DPYD activity peaks near the middle of the light span, when the animals rest [118]. In mononuclear cells of healthy subjects, and gastrointestinal or nasopharyngeal cancer patients, both *DPYD* mRNA and activity show circadian rhythms with a maximum at night (rest span), similar with mice [119,120,121]. DPYD activity was also highest at midnight in biopsies of oral mucosa in healthy subjects [122]. The opposite activity phases of DPYD and 5-FU target thymidylate synthase (TS) which is peaked at 1 p.m. in oral mucosa indicate an increased tolerability of 5-FU when administered at night [29]. Circadian rhythms in phase II detoxification by GST contribute to toxicities of platinum complexes [123]. GST catalyze the conjugation of reduced glutathione (GSH) to xenobiotics for detoxification [124]. GSH contents of liver and jejunum are nearly three-fold higher in the second half of the dark span in mice and rats as compared to mid-day, and suppression of GSH synthesis by buthionine sulfoximide greatly modifies the chronotolerance patterns of cisplatin and oxaliplatin in mice [125,126]. In summary, the circadian rhythmic changes in enzyme and transporter function may cause differences in absorption, metabolic degradation and excretion of drugs and might lead to daily bioavailability differences and unexpected outcomes. Furthermore, changing abundance of transporters and enzymes through 24-h may profoundly modify drug pharmacology/toxicology and this has potential implications for improving the tolerability of anticancer drugs. Aforementioned studies carried out by looking specific genes. To understand the effect of CTS at genome-wide level and identify biomarker genes further genomic studies require to be performed in both cancer patient and animal level.

### 3.2. Relevance of Circadian Rhythms for Chronopharmacodynamics of Anticancer Agents

ChronoPD examines the mechanisms of drug actions in relation to circadian clock and mainly deals with rhythm-dependent differences in the effects of drugs on the body, and rhythmic differences in the susceptibility or sensitivity of a biological target to drugs [53,54]. These administration time differences in the drug effects in the view of PD are due to the biological rhythms in the number and conformation of drug-specific receptors, membrane permeability, free-to-bound plasma fraction of medications, ion channel dynamics, second messengers, rate limiting steps in metabolic pathways, binding enzymatic activities and other drug targets in several molecular signal transduction pathways as well as cell cycle process [29,34,57,127,128]. For instance, dosing time-dependent anti-tumor effect of Interferon-β (IFN-β) is closely related to that of IFN receptors and interferon-stimulated gene factor (ISGF) expressions in tumor cells [129].

The CTS profoundly modifies the PD of anticancer medications in that it controls several molecular drug targets in many biological functions including cellular proliferation, cell cycle events, angiogenesis, DNA repair and apoptosis, and many signal transduction pathways which account for chronoPD in particular for anticancer drugs (see Table 3) [28,29,53,57,127]. Biological rhythms at the cellular level cause significant dosing time-dependent differences in the PD of medications, and thus may modify the efficacy and toxicity of drugs depending on the dosing time [29,112]. Mechanisms of chronotolerance and chronoefficacy involve circadian clock dependencies in anticancer drug PK, as well as circadian rhythms in relevant cellular targets in host and tumor cells. The CTS controls cyclins (*CycB1*, *CycD*, *CycE*), cyclin dependent kinases (*CDK1*, *CDK2*), cell cycle genes that gate G1/S or G2/M transitions such as *p21*, *Wee-1*, proto-oncogene *c-Myc*, DNA repair (*ATM*, *AGT*, *p53*), and apoptosis e.g., proapoptotic *Bax* and antiapoptotic *Bcl2* genes (Figure 2) [23,37,128,130,131,132].

Most of the chemotherapeutic agents are toxic for both malignant and healthy cells (bone marrow, oral mucosa, gut, and skin) in the process of cell division [34]. In addition, most of the anticancer agents show their cytotoxic effects at specific phases of the cell division cycle. For example, cells in the S-phase (during DNA synthesis) are more susceptible to anticancer drugs 5-FU [138] and irinotecan [139]. Proportions of cells in S phase and in G2/M phase increase by about 50% in the second half of the darkness period, and in contrast G0/G1 cells predominate during the light period in the bone marrow tissues in mice [56]. Best tolerated circadian time of 5-FU, irinotecan, docetaxel and gemcitabine in mice corresponds to the time when the antiapoptotic BCL2 expression is high, and proapoptotic BAX expression is low, at the early light span in mice [140,141,142,143,144]. Some of the anticancer drugs like oxaliplatin which produces DNA cross links does not have phase specificity [145]. For the optimal timing of successful cancer therapy, it is important to know the time of day when tumors reproducibly express proliferative targets relevant to cancer cell DNA synthesis or cancer cell division.

Pharmacology, and thus efficacy and toxicity of the anticancer drug 5-FU are affected by the circadian rhythms in TS which is the target enzyme of this drug and rhythms in DPYD, the rate limiting enzyme for the catabolism of 5-FU [136]. The enzyme TS catalyzes the synthesis of thymidylate, which is essential for DNA replication. The expression of *TS* gene is under the control of circadian clock, and TS activity varies throughout the day in normal human and mouse tissues in parallel with tissue S-phase fraction [135,146]. Circadian coordination of tumor TS content and activity result in daily variation in the toxic-therapeutic ratio of the TS-targeted drug 5-FU. In the study of Wood et al. [136], the best time of day (early activity, 2 h after daily arising) for 5-FU treatment to tumor-bearing mice (low host toxicity and high tumor response) was associated with the maximum daily tumor nuclear BMAL-1 protein content and total cell WEE-1 (gates cell mitosis) protein concentration, which was coincident with lowest daily average tumor size and vascular endothelial growth factor (VEGF) content as well as the lowest daily tumor (and also lowest TS activity in bone marrow and gut tissue) TS activity. Tumor TS protein content and TS activity have been used to successfully predict 5-FU tumor response in experimental and human cancers [147,148]. Wood et al. [136] used three established therapeutic targets, mitosis (cell division), TS (DNA synthesis), and VEGF (angiogenesis and growth) as pharmacological determinants of 5-FU. Cell cycle progression is tied to the circadian clock through clock-controlled WEE1, which modulates cyclin-dependent kinases. Inhibition of cyclin-dependent kinase cause cell cycle arrest and subsequent circadian stage-dependent gating of cells at G2-M interface of the cell cycle [55,149]. VEGF protein shows circadian pattern with a peak at 2–6 h after light onset [136,150] leading to variations in the in vivo response to several angiogenesis inhibitors according to the circadian time of administration. 

Antitumor effect of IFN-β in nocturnally active mice was found to be more efficient during the early rest phase than during the early active phase [129]. The higher sensitivity to tumor cells was observed when specific binding of IFN receptor and the proportion of tumor cells in S phase increased. The circadian time-dependent effect of IFN-β was also supported by the rhythmic expression of the cell proliferation inhibitor i.e., cyclin-dependent kinase (cdk) inhibitor p21 wild-type p53 fragment 1 (p21WAF1) protein induced by IFN-β [129]. In another study, the dosing time-dependent effect of imatinib mesylate on the ability of tumor growth inhibition and underlying mechanism were investigated in mice from the viewpoint of sensitivity of tumor cells to the drug [151]. Imatinib is an anticancer agent which inhibits the function of various receptors with tyrosine kinase activity, including Abl, the bcr-abl chimeric product, KIT, and platelet-derived growth factor (PDGF) receptors [152,153,154,155]. Tumor growth inhibition was greater when imatinib mesylate (50 mg/kg, i.p.) administered in the early light phase then when it was administered in the early dark phase. The dosing time-dependency of the anti-tumor efficacy of the drug was found to be related with drug-induced inhibition of PDGF receptor activity, but not with KIT or Abl. Anti-tumor activity of imatinib was increased by the delivery of the drug when PDGF receptor activity was enhanced. In other words, rhythmic changes in the tyrosine kinase activity of PDGF receptors on tumor cells seem to influence the anti-tumor efficacy of imatinib [151].

Cao et al. [156] demonstrated that mammalian target of rapamycin (mTOR) pathway was under the control of the circadian clock in the SCN in mice. Using phosphorylated S6 ribosomal protein (pS6) as a marker of mTOR activity, they showed that the mTOR cascade exhibited maximal activity during the subjective day (at CT4; Circadian Time), and minimal activity during the late subjective night [156]. A study by Okazaki et al. [157] has also reported that activity of the mTOR-signaling pathway showed circadian rhythm in renal carcinoma tumors and the 24-h rhythm of mTOR-signaling activity was regulated by protein degradation via Fbxw7 that was controlled by circadian clock systems through DBP. mTOR protein levels in both renal tumor mass and in the normal liver tissue displayed 24-h rhythms, which were higher in the dark phase than in the light phase. Improved antitumor activity was observed when everolimus was administered to tumor-bearing mice during the time of elevated mTOR activity, suggesting that high levels of mTOR activation in renal tumors rendered them more sensitive to everolimus. The survival rate of tumor-bearing mice was higher when everolimus was administered at ZT12 than administered at ZT0 [157]. In a recent preclinical study, dosing time-dependent anti-tumor effect of erlotinib and the underlying molecular mechanisms through the PI3K/AKT (phosphatidylinositol-3-kinase/protein kinase B) and ERK/MAPK (extracellular signal-regulated kinase/mitogen-activated protein kinase) pathways were investigated in nude mice HCC827 tumor xenografts models [158]. Erlotinib is a tyrosine kinase inhibitor that suppresses intracellular phosphorylation of tyrosine kinase related epidermal growth factor receptor (EGFR, whose gene is oncogene) which is a critical member of receptor tyrosine kinase family and is involved in many cancer-related signal transduction pathways including proliferation, metastasis and angiogenesis [159,160]. Rat sarcoma/rapidly accelerated fibrosarcoma/mitogen-activated protein kinase (Ras/Raf/MAPK) and PI3K/AKT are two principal pathways of EGFR downstream, which may promote mitosis and prevent apoptosis [161]. Receptor tyrosine kinase activity of EGFR, and its downstream signal pathways AKT and MAPK showed obvious 24-h oscillation [158,162]. In this study, erlotinib displayed dosing time-dependent anti-tumor activity, which was more effective on the tumor growth inhibiton when administered in the early light phase than in the early dark phase, when the activities of EGFR and its downstream factors increased. Overall, the inhibitory effect of erlotinib on phosphorylation of EGFR, AKT and MAPK changed by the dosing time, providing a clue to optimize the dosing schedule of this drug [158].

In vitro studies has also exhibited the crucial role of cellular rhythms as a major determinants of pharmacological response. Topoisomerase 1 (TOP1) is an enzyme that catalyze the breaking and rejoining of DNA strands in a way which allows the strands to pass through one another, thus altering the topology of DNA. This enzyme is the molecular target of the anticancer agent irinotecan which is active against colorectal cancer. Inhibition of this enzyme by irinotecan results in the DNA breaks and apoptosis [163]. Dulong et al. [112] reported that TOP1 enzyme showed circadian rhythms in the level of mRNA and protein in synchronized colorectal cancer cell cultures. The molecular rhythms were also found in the bioactivation, transport, metabolism, and detoxification processes of the drug leading to profound changes in the pharmacokinetics and pharmacodynamics, and resulting in a four-fold difference in irinotecan induced apoptosis according the circadian drug timing [112,113]. In this study, irinotecan cytotoxicity was found to be positively correlated with the expression level of clock gene *BMAL1*, both in clock-proficient and clock-deficient cells. In contrast, BMAL1 overexpression inhibited colorectal cancer cell proliferation and enhanced colorectal cancer sensitivity to oxaliplatin [164]. The overall survival of patients with colorectal cancer with high BMAL1 levels in their primary tumors was profoundly longer than that of patients with low BMAL1 levels (27 vs. 19 months; *p* = 0.04) [164].

### 3.3. Relevance of Circadian Rhythms for Chronotoxicity of Anticancer Agents

Many anticancer agents show severe toxicities and adverse effects including myelosuppression and reduced immune function, gastrointestinal reactions, liver and kidney dysfunction [165]. Though, chronotherapy approach cannot provide avoidance of all these adverse effects, it can alleviate unfavorable effects for achieving better compliance of patient from the cancer therapy and better treatment response [29,32,127]. The circadian timing of drug administration might be very critical in eliminating the risk of toxicity of anticancer medications, since PK and PD are influenced by the circadian rhythms in the biological functions. Studies in rodents have revealed that the dose of the drug may be lethal when given at the certain times of the day, whereas the same dose may have only little adverse effects when given at other times of the day [29]. In other words, the lethal toxicity of a fixed dose of a drug may vary as a function of dosing time. 24-h variation in drug toxicities has been documented in rodents kept in regular alternation of 12 h of light and 12 h of darkness (LD12:12) [29,32]. The tolerability of more than 40 anticancer drug may vary up to 10-fold as a function of dosing time in rodents as shown with methotrexate [117], cyclophosphamide [69], docetaxel and doxorubicin [140,141,166], vinorelbine [167], gemcitabine and cisplatin [143,168], antimetabolites 5-FU [136,144] and L-alanosine [169], irinotecan [33,142], oxaliplatin [170], seliciclib [171] and etoposide [172]. In a recently performed chronopharmacological study, a second generation nucleotide analogue clofarabine which is used for the treatment of hematological malignancies such as acute leukemia was administered to mice, and time- and dose-dependent toxicity was evaluated by examining biochemical parameters, histological changes, and organ indexes of mice. Clofarabine toxicity in mice when administered in the rest phase was more severe than the active phase, demonstrated by more severe liver damage, immunosuppression, higher mortality rate, and lower LD50 [173].

For an important issue, drug chronopharmacology and chronotoxicity usually display opposite 24-h patterns in nocturnal rodents as compared to human (day active), whose circadian physiology and molecular clock gene expression differ by almost 12 h relative to the light-dark schedule [32,54]. In a clinical study, the relationship between dosing time of gemcitabine and development of hematologic toxicity in cancer patients were revealed with the finding that gemcitabine-induced hematologic toxicity can be decreased by treating cancer patients at 9:00 [174]. The study demonstrated that treatment with gemcitabine at 9:00 decreased the toxicity on white blood cells (WBC) and platelets by nearly 10% as compared to treatment at 15:00. These findings were accompanied with the previous study results of Li et al. [143] performed in nocturnal mice which indicated that tolerability of gemcitabine was better when administered during the early active phase as compared to the late active phase. Thus, the findings of many circadian functions in rodents could be applied to humans by changing the phase of circadian regulation to the anti-phase of it. Researches on the chronopharmacology of anticancer drugs, and chronotoxicity findings in preclinical studies will benefit clinical practice in future. Hence, the undesired effects of anticancer drugs, in particularly the ones with narrow therapeutic index and high potential for adverse effects may be controlled with circadian drug timing.

## 4. Chronotherapeutics in Cancer Chemotherapy

**Figure 3:**
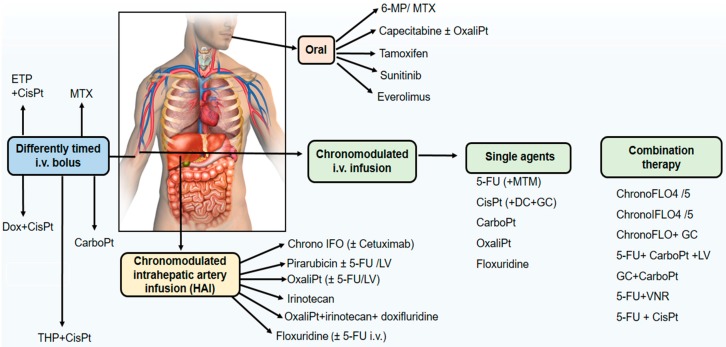
Administration route of cancer chronotherapeutics in phase II and III clinical trials. Anticancer drugs delivered by per oral, buccal, subcutaneous, intravenous (i.v.) and intrahepatic arterial (IHA) routes by using chronotherapeutic approaches. In i.v. administration routes, drugs are applied via chronomodulated fashion with different peak flow times or via i.v. bolus with different circadian time. HAI: Hepatic artery infusion; ETP: Etoposide; CisPT: Cisplatin; MTX: Methotrexate; Dox: Doxorubicin; THP: 4′-0-tetrahydropyranyl doxorubicin; CarboPT: Carboplatin; 6-MP: 6-Mercaptopurine; OxaliPt: Oxaliplatin; Chrono IFO: Chronomodulated irinotecan, 5-fluorouracil, leucovorin and oxaliplatin; LV; Leucovorin; GC: Gemcitabine; DC: Docetaxel; MTM: Mitomycin-C; ChronoFLO: Chronomodulated 5-FU, leucovorin, oxaliplatin; VNR: Vinorelbine.

Chronotherapeutics aims at improving the tolerability and efficacy of treatments, and decreasing the toxicity and undesirable side effects of medications through their delivery considering the circadian rhythms of physiological and biochemical processes. Circadian rhythms in the biological functions, and 24-h rhythm dependencies of drug PK, PD, and drug safety constitutes the rational for chronotherapy. Both the desired/beneficial and undesired/adverse effects of drugs may vary significantly depending on the dosing time. Chronotherapy is especially relevant for anticancer agents [28,29,32,127]. The activity of anticancer drugs may be restricted by their side effects, and their toxicities to healthy host tissues. Most of the antineoplastic agents show their cytotoxic effects on the cells which are at the specific phases of cell division [29]. Circadian timing of anticancer agents often results in predictable changes in drug pharmacology which may translate into clinically relevant differences in treatment outcomes. An overview of administration route of cancer chronotherapeutics in phase II and III clinical trials is presented in Figure 3.

Administration time of anticancer drugs carries importance because the CTS influences the PK of many anticancer agents and affects clinical outcomes in cancer patients [29]. Strikingly, time course of plasma concentrations of 5-fluorouracil (5-FU) in cancer patients receiving constant rate i.v. infusion for 5 days. The highest plasma concentrations of 5-FU were found around 4:00 a.m. despite constant-rate infusion [34]. Moreover, the best-tolerated time of 5-FU was also found to be 4:00 a.m. in cancer patients with a large regular circadian variation in the 5-FU plasma concentrations and a maximum plasma concentration (C_max_) located at 4:00 a.m. [29,144]. In another study, irinotecan PK and toxicity were evaluated in 31 cancer patients. Irinotecan was administered as a conventional 30-min infusion in the morning or as a chronomodulated infusion from 2:00 a.m. to 8:00 a.m. Patients receiving the chronomodulated infusion, suffered less severe diarrhea and less interpatient variability of irinotecan and SN-38 as compared to conventional therapy [175]. Recent studies also showed that daily pharmacokinetic variations were seen in estrogen receptor modulators and tyrosine kinase inhibitors in mice and cancer patients [89,176,177]. 

Since antitumor drugs may kill tumor cells as well as normal cells, the maximization of the antitumor effects and the minimization of the toxicity of anticancer agents to normal tissues are substantial in cancer chemotherapy. Chronotherapy may play a vital role in the quality of life and survival rate for cancer patients. A significant improvement in the tolerability and efficacy was demonstrated in the international randomized trials in which cancer patients were treated with the same sinusoidal chronotherapy schedule over 24-h as compared to constant rate infusion [27]. In a randomised multicentre phase III trial which enrolled 186 patients with metastatic colorectal cancer, chronomodulated infusion of oxaliplatin, 5-FU and folinic acid (ChronoFLO) administered to coincide with relevant circadian rhythm in comparison to a constant rate method of infusion. Chronomodulated delivery decreased five-fold the rate of severe mucosal toxicity as compared to constant drug delivery (14% vs. 76%), and halved that of functional impairment from the peripheral sensitive neuropathy (16% vs. 31%). Median time to treatment failure was found to be 6.4 months on chronotherapy and 4.9 months on constant rate infusion [178]. In another randomized multicenter trial, irinotecan was given as standard conventional 30-min infusion in the morning or as a chronomodulated infusion from 2:00 a.m. to 8:00 a.m., with peak delivery rate at 5:00 a.m. to 31 cancer patients with metastatic colorectal cancer. In this study, irinotecan-induced severe diarrhea episodes was fewer in chronomodulated therapy than conventional treatment [175]. Several other Phase I to Phase III clinical trials have been validated the relevance of circadian timing of anticancer medications and confirmed the efficacy of chronomodulated therapy in different kinds of tumors [179,180,181] Randomised clinical trials revealed that the gender has a prominent role for tolerability and efficacy of chronotherapeutics. In three clinical trials involving advanced or metastatic colorectal or lung cancer, chronoFLO or chronomodulated 5-FU-LV-carboplatin produced significantly more adverse events in women as compared to men [40,178]. Recent clinical studies evaluating the role of circadian timing on anticancer drug pharmacology and treatment outcomes are exemplified in Table 4.

## 5. Chronopharmaceutics and Innovative Chrono-Drug Delivery Systems in Cancer Treatment

The CTS may substantially influence the PK and PD of medications when injected, infused, inhaled or applied orally and dermally at the different circadian times of the day [38,182]. It has become indispensable to develop a drug delivery system which release the anticancer drug at the target site and appropriate time to achieve an improved progress in treatment outcomes. “Chronopharmaceutics” are new drug delivery systems which are used to synchronize drug concentrations according to circadian rhythms of the biological functions and drug pharmacology in the organism [182]. The fundamental aim of chronopharmaceutics and chrono-drug delivery systems (ChronoDDS) are to deliver drugs in the time of greatest need with the dose providing higher concentration and when the need is less providing lesser concentration for minimizing side effects and optimizing treatment outcomes of medications [29,182]. Today, the technologies available for chronopharmaceutics are programmable-in-time infusion pumps (chronomodulating infusion pumps), modified release (MR) of oral drugs, toward rhythm-sensing drug-releasing nanoparticles [38,127]. More attempts are being made for adjusting DDSs accurately to cancer patient needs, in terms of both treatment efficacy and compliance.

Conventional infusion protocols and drug formulations in the cancer treatment only consider drug doses, duration and frequency of the infusions keeping the in vivo drug concentration in the therapeutic level for a prolonged period of time. In contrast, circadian chronomodulated pulsatile drug delivery systems are characterized by a programmable lag phase after which the drug release is triggered automatically as time-controlled taking into account the rhythmic determinants in disease pathophysiology, chronopharmacology of drugs, and the CTS to identify the drug delivery pattern, dose and dosing time [38,182]. Pulsatile DDSs have the capability of releasing the drug after a predetermined lag period in pulsed or releasing in a controlled manner offering solutions for delivery of drugs which require nocturnal dosing. These systems have drawn the attention of both industry and academic research recently. Chronomodulating infusion pumps present a new infusion parameter which is called “the time of peak-flow rate” since the drug delivery pattern is not constant, i.e., it is rather semi-sinusoidal, with an increasing flow rate, a peak administration rate at a time specification and a gradual symmetric decrease in flow rate [53]. 

Chronotherapeutic approaches to drug delivery have triggered the industrial development of non-implantable multichannel programmable-in-time pumps for the treatment of cancer. Invention of the IntelliJect^TM^ device with four 30-mL reservoirs which was approved in the European Union and in North America enabled the development of first circadian chronomodulated combination delivery schedule of 5-FU, leucovorin, and oxaliplatin used safely and with increased efficacy [29,38]. Mélodie^TM^, a four-channel programmable pumps with the advantages of increased energy autonomy, flexible reservoir capacity, rapid programming of any delivery schedule and computer storage of treatment protocols and patient data. The direct administration of anticancer drugs into the hepatic artery has been shown to be beneficial in terms of both tolerability and antitumor activity on liver metastases from colorectal cancer. Since liver is one of the most rhythmic organs in the body, high differential cytotoxicity is expected from the direct chronomodulated infusion of chemotherapy into the hepatic artery that vascularizes liver metastases. Thus, liver metabolism and susceptibility are expected to be rhythmic, while liver metastases are expected to display no such robust circadian organization any more. An international trial (OPTILIV: Optimal control of liver metastases with hepatic artery infusion of 5-FU, oxaliplatin and irinotecan chemotherapy and systemic cetuximab) revealed that combination of agents by intrahepatic artery infusion elicited safe and effective therapy [183,184]. 

Chronomodulated infusions of chemotherapy have been given to non-hospitalized patients using a more recent developed elastomeric pump with programmed rhythmic electronic control of infusion flow rate, which is named CIPTM. These innovative chrono-drug delivery systems provide a chronomodulated delivery of up to four drugs over one to several days in out-patients and with minimal nursing need [29,30]. 

Recently, novel drug loaded nanocarriers have been searched for chronotherapy. Chronotherapy with drug-containing nanoformulation appears to be a new therapeutic strategy which may improve cancer curability with decreased side effects, costs, and risks for the cancer patients [185]. Chronopharmaceuticals coupled with nanotechnology could be the future of DDS, and lead to safer and more efficient disease therapy in the future [182]. In a recent study, synergistic anti-tumor efficacy of paclitaxel (PTX)-loaded polymeric nanoparticles (PTX-NPs) combined with circadian chronomodulated chemotherapy in xenografted human lung cancer was examined to screen out to best time of the day for the drug delivery [186]. The drug in PTX-NPs displayed an initial fast release and subsequently a slower and sustained release. Chronomodulated delivery of PTX-NPs exhibited greatest anti-tumor activity than that of PTX injection, and showed a time-dependent effect which was the best at 15 h after light onset, suggesting a potential treatment for lung cancer [186]. The synergistic mechanism was suggested to be related to the inhibition of tumor cell proliferation through its action on Ki-67, and decreased microvessel density associated with CD31 and promoted cell apoptosis [186]. New chronopharmaceutical technologies are underway of development and testing for delivering anticancer agents precisely in a circadian time-dependent manner by bedside or ambulatory pumps for cancer treatment.

## 6. Conclusions and Perspectives

Chronotherapy has become an intriguing research subject in recent years particularly in cancer, rheumatology, neurological/psychiatric disorders, and cardiovascular diseases. However, chronotherapeutic approaches do not reach to the desired level in the pharmacotherapy. Pharmaceutical industry, local drug authorities and clinicians should consider the effects of circadian rhythms on the efficacy and/or tolerability of drugs, and drug dosing time which is a critical factor should not be underestimated. The clinicians should more focus on this issue and perform new clinical trials to show benefit of chronotherapy in cancer, cardiovascular and other diseases.

The circadian rhythms in the biological functions and circadian physiology, clock genes as well as cell cycle events may substantially affect the cancer chemotherapy depending on the drug delivery schedule. Yet, many factors including gender, age, physical activity, disease states, genetic polymorphism and phenotypic properties of an individual result in the inter-individual and intra-individual differences in drug exposure and pharmacokinetic parameters leading to a large variability in the therapeutic response [27,29,194]. 

To establish a rational chronotherapeutic strategy for choosing the appropriate dosing time, it is compulsory to identify the CTS status and predict optimal treatment timing in cancer patients. With respect to this translational point, Ortiz-Tudela and co-workers proposed monitoring of temperature, activity and position of metastatic gastrointestinal cancer patients by employing inner wrist surface Temperature, arm Activity and Position (TAP) [195]. Pursuing of circadian biomarkers seems smart approach but clinical validation is necessary in that inter- and intra-individual changes for these parameters. Identification of the potential rhythmic key determinants of anticancer drug chronopharmacology associated with the drug metabolism and detoxification, molecular drug targets and their molecular clock control are necessary for optimally shaping the circadian drug delivery patterns to individual CTS in cancer patients. CYCLOPS i.e., cyclic ordering by periodic structure is very recently proposed algorithm by Anafi and coworkers [196]. To develop the algorithm, large human liver and lung biopsy as well as mouse tissue samples were collected and rhythmic transcripts of many drug targets and disease gene were identified. In vivo validation of the method was carried out by using streptozocin which destroys pancreatic beta cells. The authors showed that the dosage time can temporally segregate efficacy from dose-limiting toxicity of streptozocin. The TAP approach and CYCLOPS algorithm will enable us to assess the effectiveness and safety of the chemotherapy for individual patients, which bring a personalized chrono-chemotherapy.

We may claim that randomized clinical trials employed chronotherapy for cancer are limited as compared to other clinical studies. Further studies should be performed for better understanding of clinical outcomes of cancer chronotherapy in particular women cancer patients. Non-invasive and clinically validated techniques for determining the CTS status, data based mathematical modelling, system chronopharmacological and data based translational approaches will help achieving better efficacy and acceptable adverse effect of drugs in particular anticancer agents. New chronopharmaceutical and chrono-drug delivery products enable to clinicians for chronotherapeutic applications in cancer and other diseases. 

## Figures and Tables

**Table 1:** This table summarizes the amount and activities of CYP 450 level at both molecular and biochemical levels in various circadian times in mammals along with rhythm parameters from different studies.

Enzyme	Parameter	Method	Tissue/Cell Line	Species	Strain	Sex	Age	Peak	Trough	Cosinor	ANOVA	Reference
Cyp11a1	mRNA	RT-PCR	Adrenal gland	Mouse	*WT*	-	-	ZT0	ZT12	-	-	[81]
Cyp11b1	mRNA	RT-PCR	Adrenal gland	Mouse	*Crem KO*	-	-	ZT0	ZT12	-	-	[81]
Cyp17	mRNA	Microarray	Liver	Mouse	*CD-1*	Male	Mature	ZT14-18	ZT2	-	-	[82]
Cyp17a1	mRNA	RT-PCR	Adrenal gland	Mouse	*Crem KO*	-	-	ZT12	ZT0	-	-	[81]
Cyp1a1	mRNA	RT-PCR	Liver	Mouse	*Kunming*	Male	8 W	ZT6	ZT18-22	*p* < 0.05	-	[75]
Cyp1a1	mRNA	RT-PCR	Liver	Mouse	*Kunming*	Female	8 W	ZT6	ZT22	*p* < 0.05	-	[75]
Cyp1a1	mRNA	RT-PCR	Lung	Mouse	*WT*	-	-	ZT14	ZT6	-	*p* < 0.05	[83]
Cyp1a1	Protein	Western blot	Lung	Mouse	*WT*	-	-	ZT18	ZT6	-	-	[83]
Cyp1a1	Activity	P450-Glo kit	Lung	Mouse	*WT*	-	-	ZT18	ZT2	-	*p* < 0.05	[83]
Cyp1a2	mRNA	RT-PCR	Liver	Mouse	*C57BL/6*	Male	8 W	ZT13	ZT1	-	-	[84]
Cyp1a2	mRNA	RT-PCR	Liver	Mouse	*Kunming*	Male	8 W	ZT6	ZT18-22	*p* < 0.05	-	[75]
Cyp1a2	mRNA	RT-PCR	Liver	Mouse	*Kunming*	Female	8 W	ZT6	ZT22	*p* < 0.05	-	[75]
Cyp21a1	mRNA	RT-PCR	Adrenal gland	Mouse	*WT*	-	-	ZT12	ZT0	-	-	[81]
Cyp26a1	mRNA	RT-PCR	Liver	Mouse	*ICR*	Male	6 W	ZT6-10	ZT2	-	-	[85]
Cyp27a1	mRNA	RT-PCR	Liver	Mouse	*Kunming*	Male	8 W	ZT10	ZT22	*p* < 0.05	-	[75]
Cyp27a1	mRNA	RT-PCR	Liver	Mouse	*Kunming*	Female	8 W	ZT10	ZT22	*p* < 0.05	-	[75]
Cyp2a1	Activity	THA	Testis	Rat	*Fischer 344*	Male	18 W	ZT8	ZT20	-	-	[86]
Cyp2a4	mRNA	Microarray	Liver	Mouse	*CD-1*	Male	Mature	ZT14-18	ZT2	-	-	[82]
Cyp2a4	mRNA	RPA	Liver	Mouse	*129/Ola*	Male	10–16 W	ZT11-15	ZT23	-	-	[78]
Cyp2a4	mRNA	RT-PCR	Liver	Mouse	*Kunming*	Male	8 W	ZT10	ZT22	*p* < 0.05	-	[75]
Cyp2a5	mRNA	RPA	Liver	Mouse-NTF	*-*	-	10–16 W	ZT13	ZT0	-	-	[87]
Cyp2a5	mRNA	RPA	Liver	Mouse-DTF	*-*	-	10–16 W	ZT0	ZT12	-	-	[87]
Cyp2a5	mRNA	ADDER	Liver	Mouse-NTF	*-*	-	10–16 W	ZT16	ZT4	-	-	[87]
Cyp2a5	mRNA	ADDER	Liver	Mouse-DTF	*-*	-	10–16 W	ZT4	ZT16	-	-	[87]
Cyp2a5	mRNA	RBA	Liver	Mouse	*129/Ola*	Male	10–16 W	ZT11-15	ZT23-3	-	-	[78]
Cyp2b10	mRNA	Northern blot (PTB induced)	Liver	Mouse	*-*	-	-	ZT16	ZT4	-	-	[66]
Cyp2b10	mRNA	Northern blot (PTB induced)	Intestine	Mouse	*-*	-	-	ZT16	ZT4	-	-	[66]
Cyp2b10	mRNA	RT-PCR	Liver	Mouse	*Kunming*	Male	8 W	ZT14-18	ZT22-2	*p* < 0.05	-	[75]
Cyp2b10	mRNA	RT-PCR	Liver	Mouse	*Kunming*	Female	8 W	ZT18	ZT6	*p* < 0.05	-	[75]
Cyp2b10	mRNA	Branched DNA assay	Liver	Mouse	*C57BL/6*	Male	9 W	ZT21	ZT9	-	-	[60]
Cyp2c11	mRNA	RT-PCR	Hippocampus	Rat	*Wistar*	Male	-	ZT2.58	-	-	*p* = 0.0017	[88]
Cyp2c11	mRNA	RT-PCR	Middle cerebral artery	Rat	*Wistar*	Male	-	ZT5.11	-	-	*p* = 0.025	[88]
Cyp2c11	mRNA	RT-PCR	Iinferior vena cava	Rat	*Wistar*	Male	-	ZT4.20	-	-	*p* = 0.011	[88]
Cyp2c50	pre-mRNA	RT-PCR	Liver	Mouse	*-*	-	-	ZT20	ZT4	-	-	[66]
Cyp2d10	mRNA	RT-PCR	Liver	Mouse	*FVB*	Female	8–12 W	ZT20	ZT8	*p* < 0.0413	-	[89]
Cyp2d22	mRNA	RT-PCR	Liver	Mouse	*FVB*	Female	8–12 W	ZT20	ZT8	*p* < 0.0413	-	[89]
Cyp2e1	mRNA	Microarray	Liver	Mouse	*CD-1*	Male	Mature	ZT14-18	ZT2	-	-	[82]
Cyp2e1	Activity	*p*-Nitrophenol hydroxylation	Liver	Rat	*Sprague Dawley*	Male	-	ZT12	ZT0	-	-	[90]
Cyp2e1	mRNA	RT-PCR	Liver	Rat	*Wistar*	Male	8 W	ZT12	ZT21	-	*p* < 0.05	[91]
Cyp2e1	Protein	Western blot	Liver	Rat	*Wistar*	Male	8 W	ZT18	ZT3	-	*p* < 0.01	[91]
Cyp2e1	Activity	Hydroxylation/HPLC	Liver	Rat	*Wistar*	Male	8 W	ZT21	ZT3	-	*p* < 0.05	[91]
Cyp2e1	mRNA	RT-PCR	Kidney	Rat	*Wistar*	Male	8 W	ZT12	-	-	-	[91]
Cyp2e1	Protein	Western blot	Kidney	Rat	*Wistar*	Male	8 W	ZT21	ZT6	-	*p* < 0.05	[91]
Cyp2e1	Activity	Hydroxylation/HPLC	Kidney	Rat	*Wistar*	Male	8 W	ZT21	ZT6	-	*p* < 0.05	[91]
Cyp2e1	mRNA	RT-PCR	Liver	Mouse	*Kunming*	Male	8 W	ZT10	ZT22	*p* < 0.05	-	[75]
Cyp2e1	mRNA	RT-PCR	Liver	Mouse	*Kunming*	Female	8 W	ZT10	ZT22	*p* < 0.05	-	[75]
Cyp2e1	Activity	*p*-Nitrophenol hydroxylation	Liver	Mouse-*AD*	*ICR*	Male	7 W	ZT14-18	ZT2-6	-	*p* < 0.01	[92]
Cyp2e1	Activity	*p*-Nitrophenol hydroxylation	Liver	Mouse-TRF	*ICR*	Male	7 W	ZT22-2	ZT14	-	*p* < 0.01	[92]
Cyp2e1	mRNA	Branched DNA assay	Liver	Mouse	*C57BL/6*	Male	9 W	ZT17	ZT5	-	-	[60]
Cyp3a	Activity	EDA	Liver	Rat	*Wistar*	Male	10 W	ZT19	ZT6	-	-	[93]
Cyp3a	Activity	EDA	Liver	Rat	*Wistar*	Male	22 W	ZT19	ZT8	-	-	[93]
Cyp3a11	mRNA	RT-PCR	Small intestine	Mouse	*FVB*	Female	8–12 W	ZT16	ZT4	*p* < 0.0172	-	[89]
Cyp3a11	mRNA	RT-PCR	Liver	Mouse	*ICR*	Male	6 W	ZT10	ZT22	-	-	[85]
Cyp3a11	mRNA	RT-PCR	Liver	Mouse	*Kunming*	Male	8 W	ZT2	ZT14-16	*p* < 0.05	-	[75]
Cyp3a11	mRNA	RT-PCR	Liver	Mouse	*Kunming*	Female	8 W	ZT2	ZT14-22	*p* < 0.05	-	[75]
Cyp3a11	mRNA	Branched DNA assay	Liver	Mouse	*C57BL/6*	Male	9 W	ZT21	ZT1	-	-	[60]
Cyp3a25	mRNA	RT-PCR	Liver	Mouse	*Kunming*	Male	8 W	ZT2	ZT14-16	*p* < 0.05	-	[75]
Cyp3a25	mRNA	RT-PCR	Liver	Mouse	*Kunming*	Female	8 W	ZT2	ZT14-22	*p* < 0.05	-	[75]
Cyp4a10	mRNA	RT-PCR	Liver	Mouse	*Kunming*	Male	8 W	ZT10	ZT22	*p* < 0.05	-	[75]
Cyp4a10	mRNA	RT-PCR	Liver	Mouse	*Kunming*	Female	8 W	ZT18	ZT6-10	*p* < 0.05	-	[75]
Cyp4a14	mRNA	RT-PCR	Liver	Mouse	*Kunming*	Male	8 W	ZT10	ZT22	*p* < 0.05	-	[75]
Cyp4a14	mRNA	RT-PCR	Liver	Mouse	*Kunming*	Female	8 W	ZT10	ZT22	*p* < 0.05	-	[75]
Cyp4a14	mRNA	Branched DNA assay	Liver	Mouse	*C57BL/6*	Male	9 W	ZT13	ZT21	-	-	[60]
Cyp4x1	mRNA	RT-PCR	Hippocampus	Rat	*Wistar*	Male	-	ZT8.24		-	*p* = 0.0016	[88]
Cyp4x1	mRNA	RT-PCR	Inferior vena cava	Rat	*Wistar*	Male	-	ZT18.58		-	*p* = 0.0003	[88]
Cyp51	mRNA	RT-PCR	Adrenal gland	Mouse	*WT*		-	ZT16	ZT4	-	-	[81]
Cyp51	mRNA	Northern blot	Liver	Rat	*Wistar*	Male	8 W	ZT14-22	ZT10	-	-	[94]
Cyp7a	mRNA	in situ hybridization	Liver	Rat	*Fischer*	Male	-	ZT16	ZT4	-	-	[95]
Cyp7a	mRNA	RT-PCR	Liver	Rat	*Wistar*	Male	8 W	ZT16	ZT4	-	-	[96]
Cyp7a	mRNA	Northern blot	Liver	Rat	*Wistar*	Male	8 W	ZT18	ZT6	-	-	[94]
Cyp7a	Activity	7α-hydroxylase	Liver	Rat-*AD*	*Wistar*	Male	-	ZT14	ZT6	-	*p* < 0.05	[97]
Cyp7a	Activity	7α-hydroxylase	Liver	Rat-TRF	*Wistar*	Male	-	ZT7	ZT19	-	*p* < 0.05	[97]
Cyp7a	mRNA	Northern blot	Liver	Rat-*AD*	*Wistar*	Male	-	ZT14	ZT7	-	*p* < 0.01	[97]
Cyp7a	mRNA	Northern blot	Liver	Rat-TRF	*Wistar*	Male	-	ZT7	ZT14	-	*p* < 0.01	[97]
Cyp7a1	Activity	Enzymatic	Liver	Rat	*Wistar*	Male	-	ZT16	ZT0-4	-	-	[98]
Cyp7a1	mRNA	RT-PCR	Liver	Rat-*AD*	*F344/DuCrj*	Male	7 W	ZT18	ZT6	-	*p* < 0.01	[99]
Cyp7a1	mRNA	RT-PCR	Liver	Rat-TRF	*F344/DuCrj*	Male	7 W	ZT6	ZT18	-	*p* < 0.01	[99]
Cyp7a1	mRNA	RPA	Liver	Rat	*Lewis*	-	-	ZT16	ZT4	-	-	[100]
Cyp7a1	mRNA	RT-PCR	Liver	Mouse	*Kunming*	Male	8 W	ZT10	ZT22	*p* < 0.05	-	[75]
Cyp7a1	mRNA	RT-PCR	Liver	Mouse	*Kunming*	Female	8 W	ZT10	ZT22	*p* < 0.05	-	[75]
Cyp7a1	mRNA	Northern blot	Liver	Rat	*Wistar*	Male	-	10 p.m.	10 a.m.	-	-	[101]
Cyp7a1	Protein	Western blot	Liver	Rat	*Wistar*	Male	-	10 p.m.	10 a.m.	-	-	[101]
Cyp7a1	Activity	Hydroxylase	Liver	Rat	*Wistar*	Male	-	10 p.m.	10 a.m.	-	-	[101]
Cyp7b1	mRNA	RT-PCR	Liver	Mouse	*Kunming*	Female	8 W	ZT6	ZT22	*p* < 0.05	-	[75]
Cyp8b	mRNA	RT-PCR	Liver	Rat	*Wistar*	Male	8 W	ZT7-10	ZT19	-	-	[96]
Cyp8b	mRNA	Northern blot	Liver	Rat	*Wistar*	Male	8 W	ZT10	ZT22	-	-	[94]
Cyp8b	Activity	12α-hydroxylase	Liver	Rat-*AD*	*Wistar*	Male	-	ZT14	ZT6	-	*p* < 0.05	[97]
Cyp8b	Activity	12α-hydroxylase	Liver	Rat-TRF	*Wistar*	Male	-	ZT7	ZT19	-	*p* < 0.05	[97]
Cyp8b	mRNA	Northern blot	Liver	Rat-*AD*	*Wistar*	Male	-	ZT7	ZT19	-	*p* < 0.01	[97]
Cyp8b	mRNA	Northern blot	Liver	Rat-TRF	*Wistar*	Male	-	ZT19	ZT14	-	*p* < 0.05	[97]
P450	Protein	from [102]	Liver	Rat-*AD*	*F344*	-	10 W	ZT3	ZT19	-	*p* < 0.01	[103]
P450	Protein	from [102]	Liver	Rat-fasted	*F344*	-	10 W	ZT19-23	ZT7	-	*p* < 0.01	[103]
P450	Activity	7ACoD	Liver	Rat	*F344*	-	10 W	ZT19-23	ZT7	-	*p* < 0.01	[103]
P450	Activity	7ACoD	Liver	Rat-*AD*	*F344/DuCrj*	Male	7 W	ZT18	ZT6	-	*p* < 0.01	[99]
P450	Activity	7ACoD	Liver	Rat-TRF	*F344/DuCrj*	Male	7 W	ZT6	ZT18	-	*p* < 0.01	[99]

ZT, Zeitgeber Time; RT-PCR, reverse transcription-polymerase chain reaction; WT, wild type; KO, knock-out; THA, Testosterone hydroxylase activity; ADDER, amplification of double-stranded cDNA end restriction fragments; RPA, Ribonuclease Protection Assay; AD, ad libitum; TRF, time-restricted feeding; NTF, night time feeding; DTF, day time feeding; 7ACoD, 7-alkoxycoumarin o-dealkylase; EDA, Erythromycin N-demethylase Activity; PTB, pentobarbital; W, week; Y, year. (-), means “not mentioned”.

**Table 2:** This table summarizes the amount and activities of ABC transporter level at both molecular and biochemical levels in various circadian times in mammals along with rhythm parameters from different studies.

Transporter	Parameter	Method	Tissue/Cell Line	Species	Strain	Sex	Age	Peak	Trough	Cosinor	ANOVA	Reference
Abcb1	mRNA	RT-PCR	Small intestine	Monkey	*Cynomolgus*	Male	4–7 Y	ZT3	ZT9	-	NS	[108]
Abcb1	mRNA	RT-PCR	Liver	Monkey	*Cynomolgus*	Male	4–7 Y	ZT21	ZT15	-	NS	[108]
Abcb1a	mRNA	RT-PCR	Liver	Mouse	*C57BL/6*	Male	10 W	ZT12-16	ZT0	-	*p* < 0.01	[109]
Abcb1a	mRNA	RT-PCR	Jejunum	Mouse	*C57BL/6*	Male	10 W	ZT12	ZT0	-	*p* < 0.01	[109]
Abcb1a	mRNA	RT-PCR	Kidney	Mouse	*C57BL/6*	Male	10 W	ZT12	ZT0	-	NS	[109]
Abcb1a	mRNA	RT-PCR	Ileum	Mouse	*B6D2F1*	Female	10 W	ZT15	ZT3	-	NS	[33]
Abcb1a	mRNA	RT-PCR	Jejunum (proximal)	Rat-*AD*	*Wistar*	Male	8 W	ZT12	ZT0	-	*p* < 0.05	[105]
Abcb1a	mRNA	RT-PCR	Jejunum (proximal)	Rat-TRF	*Wistar*	Male	8 W	ZT0	ZT12	-	*p* < 0.01	[105]
Abcb1a	Activity	Digoxin conc./intestinal perfusion	Jejunum (proximal)	Rat-*AD*	*Wistar*	Male	8 W	ZT18	ZT6	-	*p* < 0.05	[105]
Abcb1a	Activity	Digoxin AUC/intestinal perfusion	Jejunum (proximal)	Rat-*AD*	*Wistar*	Male	8 W	ZT18	ZT6	-	*p* < 0.01	[105]
Abcb1a	Activity	Digoxin conc.	Jejunum (proximal)	Rat-TRF	*Wistar*	Male	8 W	ZT6	ZT18	-	*p* < 0.05	[105]
Abcb1a	Activity	Digoxin AUC	Jejunum (proximal)	Rat-TRF	*Wistar*	Male	8 W	ZT6	ZT18	-	*p* < 0.05	[105]
Abcb1a	mRNA	RT-PCR	Ileum	Mouse	*C57BL/6*	Male	-	ZT10	ZT2	-	-	[67]
Abcb1a	mRNA	RT-PCR	Liver	Mouse	*C57BL/6*	-	8 W	ZT13	ZT1	*p* = 0.016	*p* = 0.029	[111]
Abcb1a/1b	mRNA	RT-PCR	Jejunal mucosa	Rat	*SD*	Male	-	ZT6	ZT18	*p* < 0.05	*p* < 0.0001	[107]
Abcb1a/1b	mRNA	Branched DNA assay	Liver	Mouse	*C57BL76*	Male	9 W	ZT16	ZT0	-	-	[60]
Abcb1b	mRNA	RT-PCR	Liver	Mouse	*C57BL/6*	Male	10 W	ZT16	ZT0	-	NS	[109]
Abcb1b	mRNA	RT-PCR	Jejunum	Mouse	*C57BL/6*	Male	10 W	ZT20	ZT0	-	NS	[109]
Abcb1b	mRNA	RT-PCR	Kidney	Mouse	*C57BL/6*	Male	10 W	ZT20	ZT0	-	NS	[109]
Abcb1b	mRNA	RT-PCR	Ileum	Mouse	*B6D2F1*	Female	10 W	ZT15	ZT3	-	*p* = 0.03	[33]
ABCB1	mRNA	RT-PCR	Caco-2	Human	*-*	-	-	ZT16	ZT4	*p* = 0.013	-	[112]
ABCB1	mRNA	RT-PCR	Caco-2	Human	*-*	-	-	ZT16	ZT4	-	-	[113]
abcb1a	mRNA	RT-PCR	AdenoCA colon 26	Mouse	*-*	-	-	ZT0	ZT12	-	-	[67]
P-gp	Protein	Western blot	Liver	Mouse	*C57BL/6*	-	8 W	ZT20	ZT0	NS	-	[111]
P-gp	Protein	Western blot	Liver	Mouse	*C57BL/6*	Male	10 W	ZT8	ZT20	-	NS	[109]
P-gp	Protein	Western blot	Kidney	Mouse	*C57BL/6*	Male	10 W	ZT0	ZT16	-	NS	[109]
P-gp	Protein	Western blot	Jejunum	Mouse	*C57BL/6*	Male	10 W	ZT8	ZT0	-	*p* = 0.04	[109]
P-gp	Activity	Digoxin accumulation	Jejunum	Mouse	*C57BL/6*	Male	10 W	ZT12	ZT0	-	*p* < 0.05	[109]
P-gp	Protein	Western blot	Jejunum	Monkey	*Cynomolgus*	Male	4–7 Y	ZT21	ZT9	-	NS	[108]
P-gp	Activity	Quinidine conc.	Brain homogenate	Rat	*Wistar*	Male	-	ZT8	ZT20	-	*p* < 0.05	[114]
P-gp	Activity	Unbound quinidine in CSF	Microdialysis	Rat	*Wistar*	Male	-	ZT8	ZT20	-	*p* < 0.05	[114]
P-gp	Protein	Western blot	Ileum	Mouse	*C57BL/6*	Male	-	ZT10	ZT2	-		[67]
P-gp	Activity	Talinolol intestinal perfusion	Jejunum	Rat-fasted	*Wistar*	Male	-	ZT13-15	ZT1-3	-	*p* < 0.05	[115]
P-gp	Activity	Talinolol intestinal perfusion	Ileum	Rat-fasted	*Wistar*	Male	-	ZT13-15	ZT1-3	-	*p* < 0.05	[115]
P-gp	Activity	Losartan intestinal perfusion	Jejunum	Rat-fasted	*Wistar*	Male	-	ZT13-15	ZT1-3	-	*p* < 0.05	[115]
P-gp	Activity	Losartan intestinal perfusion	Ileum	Rat-fasted	*Wistar*	Male	-	ZT13-15	ZT1-3	-	*p* < 0.05	[115]
P-gp	Activity	[18F]MC225 PET	Whole brain	Rat	*SD*	Male	14–16 W	ZT3-9	ZT15	-	*p* < 0.001	[116]
P-gp	Activity	[18F]MC225 PET	Cortex	Rat	*SD*	Male	14–16 W	ZT3-9	ZT15	-	*p* < 0.001	[116]
P-gp	Activity	[18F]MC225 PET	Striatum	Rat	*SD*	Male	14–16 W	ZT3	ZT15	-	*p* < 0.01	[116]
P-gp	Activity	[18F]MC225 PET	Hippocampus	Rat	*SD*	Male	14–16 W	ZT3	ZT15	-	*p* < 0.01	[116]
P-gp	Activity	[18F]MC225 PET	Cerebellum	Rat	*SD*	Male	14–16 W	ZT3-9	ZT15	-	*p* < 0.01	[116]
P-gp	Activity	[18F]MC225 PET	Pons	Rat	*SD*	Male	14–16 W	ZT3-9	ZT15	-	*p* < 0.01	[116]
Abcb4	mRNA	RT-PCR	Liver	Mouse	*C57BL/6*	Male	10 W	ZT8	ZT20	-	*p* = 0.06	[109]
Abcb4	mRNA	RT-PCR	Jejunum	Mouse	*C57BL/6*	Male	10 W	ZT4	ZT16	-	NS	[109]
Abcb4	mRNA	RT-PCR	Kidney	Mouse	*C57BL/6*	Male	10 W	ZT0	ZT12	-	NS	[109]
Abcb4	mRNA	Northern blot	Liver	Mouse	*Slc:ICR*	Male	-	ZT0	ZT16	-	-	[106]
Abcb4	mRNA	Branched DNA assay	Liver	Mouse	*C57BL/6*	Male	9 W	ZT4	ZT16	-	-	[60]
ABCC1	mRNA	RT-PCR	Caco-2	Human	*-*	-	-	ZT10	ZT0	-	-	[113]
ABCC2	mRNA	RT-PCR	Caco-2	Human	*-*	-	-	ZT12	ZT0	-	-	[113]
Abcc2	mRNA	RT-PCR	Liver	Mouse	*C57BL/6*	Male	10 W	ZT12	ZT0	-	*p* < 0.01	[109]
Abcc2	mRNA	RT-PCR	Jejunum	Mouse	*C57BL/6*	Male	10 W	ZT8	ZT20	-	*p* < 0.01	[109]
Abcc2	mRNA	RT-PCR	Kidney	Mouse	*C57BL/6*	Male	10 W	ZT12	ZT0	-	*p* = 0.02	[109]
Abcc2	mRNA	RT-PCR	Liver	Mouse	*C57BL/6*	-	8 W	ZT7	ZT19	*p* = 0.032	*p* = 0.045	[111]
Abcc2	Protein	Western blot	Liver	Mouse	*C57BL/6*	-	8 W	ZT16	ZT4	*p* < 0.05		[111]
Abcc2	mRNA	RT-PCR	Ileum mucosa	Mouse	*B6D2F1*	Male	-	ZT12	ZT0	*p* = 0.0023	*p* = 0.04	[31]
Abcc2	mRNA	RT-PCR	Ileum mucosa	Mouse	*B6D2F1*	Female	-	ZT9	ZT0	*p* = 0.0023	*p* = 0.008	[31]
Abcc2	mRNA	RT-PCR	Ileum mucosa	Mouse	*B6CBAF1*	Male	-	ZT12	ZT0	*p* = 0.00026	*p* = 0.004	[31]
Abcc2	mRNA	RT-PCR	Ileum mucosa	Mouse	*B6CBAF1*	Female	-	ZT9	ZT0	*p* = 0.00012	*p* = 0.004	[31]
Abcc2	mRNA	RT-PCR	Ileum serosa	Mouse	*B6D2F1*	Male	-	ZT4	ZT20	NS	NS	[31]
Abcc2	Protein	IHC	Ileum mucosa	Mouse	*B6D2F1*	Male	-	ZT12	ZT15	-	*p* < 0.001	[31]
Abcc2	Protein	IHC	Ileum mucosa	Mouse	*B6D2F1*	Female	-	ZT12	ZT3	-	*p* < 0.001	[31]
Abcc2	Protein	IHC	Ileum mucosa	Mouse	*B6CBAF1*	Male	-	ZT15	ZT12	-	*p* < 0.001	[31]
Abcc2	Protein	IHC	Ileum mucosa	Mouse	*B6CBAF1*	Female	-	ZT0	ZT15	-	*p* < 0.001	[31]
Abcc2	mRNA	RT-PCR	Jejunal mucosa	Rat	*SD*	Male	-	ZT12	ZT3	*p* < 0.05	*p* = 0.001	[107]
Abcc2	mRNA	Branched DNA assay	Liver	Mouse	*C57BL/6*	Male	9 W	ZT4	ZT16	-	-	[60]
MRP-2	Protein	Western blot	Jejunum	Monkey	*Cynomolgus*	Male	4–7 Y	ZT21	ZT9	-	NS	[108]
Abcg2	mRNA	RT-PCR	Liver	Mouse	*Per1 and Per2 knockout*	-	8 W	ZT7	ZT19	*p* = 0.023	*p* = 0,049	[111]
Abcg2	mRNA	RT-PCR	Jejunal mucosa	Rat	*SD*	Male	-	ZT3	ZT15	*p* < 0.05	*p* = 0.04	[107]
Abcg2	mRNA	Branched DNA assay	Liver	Mouse	*C57BL/6*	Male	9 W	ZT16	ZT4	-	-	[60]
Abcg2 isoform B	mRNA	RT-PCR	Liver	Mouse	*ICR*	-	-	ZT6	ZT18	-	*p* < 0.05	[104]
Abcg2 isoform B	mRNA	RT-PCR	Kidney	Mouse	*ICR*	-	-	ZT10	ZT18	-	*p* < 0.05	[104]
Abcg2 isoform B	mRNA	RT-PCR	Small intestine	Mouse	*ICR*	-	-	ZT6	ZT22	-	*p* < 0.05	[104]
ABCG2	Protein	Western blot	Jejunum	Monkey	*Cynomolgus*	Male	4–7 Y	ZT15-21	ZT9	-	NS	[108]
ABCG2	mRNA	RT-PCR	Caco-2	Human	-	-	-	ZT12	ZT0	-	-	[113]
abcg2	mRNA	RT-PCR	aMoS7	Mouse	-	-	-	ZT12	ZT0	-	-	[104]

ZT, Zeitgeber Time; NS, not significant; RT-PCR, reverse transcription-polymerase chain reaction; AUC, area under the curve; CSF, cerebrospinal fluid; PET, positron emission tomography; Caco-2, human colon carcinoma cell line; aMoS7, Immortalized small intestine epithelial cells; AdenoCA, adenocarcinoma; *AD*, ad libitum; TRF, Time restricted feeding; IHC, immunohistochemistry; SD, Sprague Dawley; W, week; Y, year. (-), means “not mentioned”.

**Table 3:** The targets of the anticancer drugs that are under the control of circadian clock.

Target	Parameter	Method	Tissue	Species	Strain	Sex	Peak	Trough	Cosinor	ANOVA	Reference
BCL2	Protein	Western blot	Bone marrow	Mouse	*C3H/HeN*	Male	ZT3	ZT15	*p* = 0.024	-	[56]
BCL2	Protein	Western blot	Bone marrow	MA13/C bearing mouse	*C3H/HeN*	Male	ZT7	ZT23	*p* = 0.001	-	[56]
BCL2	Protein	Western blot	Bone marrow	Mouse	*B6D2F1*	Male	ZT4–7	ZT1	*p* = 0.025	-	[56]
c-Myc	mRNA	Northern blot	Liver	Mouse	*129/C57BL6*	-	ZT14	ZT10	-	-	[133]
Cdk-4	mRNA	Northern blot	Liver	Mouse	*129/C57BL6*	-	ZT18	ZT10	-	-	[133]
Cyclin D1	mRNA	Northern blot	Liver	Mouse	*129/C57BL6*	-	ZT14	ZT22	-	-	[133]
Cyclin A (G2)	Protein	IHC	Oral mucosa	Human	-	Male	ZT20	ZT4	*p* < 0.001	-	[134]
Cyclin B1 (M)	Protein	IHC	Oral mucosa	Human	-	Male	ZT20	ZT12	*p* = 0.016	-	[134]
Cyclin E (G1/S)	Protein	IHC	Oral mucosa	Human	-	Male	ZT16	ZT4	*p* < 0.001	-	[134]
p53 (G1)	Protein	IHC	Oral mucosa	Human	-	Male	ZT12	ZT0	*p* = 0.016	-	[134]
TS	Activity	Tritium release assay	Oral mucosa	Human	-	Male	ZT16	ZT4	*p* = 0.008	NS	[135]
TS	Activity	Tritium release assay	Bone marrow	Mouse	*CD2F1*	Female	ZT18	ZT2	*p* < 0.001	*p* < 0.001	[136]
TS	Activity	Tritium release assay	Small intestine	Mouse	*CD2F1*	Female	ZT6	ZT18	*p* < 0.001	*p* < 0.001	[136]
TS	Activity	Tritium release assay	Tumor	Mouse	*CD2F1*	Female	ZT6	ZT14	*p* = 0.003	*p* < 0.001	[136]
TS	mRNA	PCR	Tumor	Mouse	*CD2F1*	Female	ZT2	ZT14	NS	NS	[136]
TS	Protein	Western blot	Tumor	Mouse	*CD2F1*	Female	ZT22	ZT14	*p* < 0.001	*p* = 0.008	[136]
Wee-1	Protein	Western blot	Tumor	Mouse	*CD2F1*	Female	ZT14	ZT18	*p* = 0.011	*p* = 0.047	[136]
Top-1	mRNA	RT-PCR	Liver	Mouse	*ICR*	Male	ZT21	ZT9	-	-	[137]
Top-1	mRNA	RT-PCR	Tumor	Mouse	*ICR*	Male	ZT21	ZT9	-	-	[137]
Top-1	Activity	Relaxation of SCP DNA	Sarcoma 180 tumor	Tumor bearing mouse	*ICR*	Male	ZT5	ZT13	-	*p* < 0.05	[137]

ZT, Zeitgeber Time; Cdk-4, cyclin-dependent kinase-4; PCR, polymerase chain reaction; IHC, Immunohistochemistry; Top-1, Topoisomerase-1 enzyme; TS, Thymidylate synthase; MA13/C, Mammary adenocarcinoma; NS, not significant; SCP, Supercoiled plasmid. (-), means “not mentioned”.

**Table 4:** Examples from the literature on relationship between circadian clock and cancer treatment at clinical level.

Anticancer Drug(s)	Cancer Type	Study Design	Dose/Chronomodulated Schedule	Main Pharmacological Findings	Main Clinical Findings	Reference
				Pharmacokinetics/Pharmacodynamics	Efficacy/Toxicity/Adverse Effects	
Cisplatin (combined with Gemcitabine and Docetaxel)	Non-small cell lung cancer	Randomized controlled study, pharmacokinetic analysis	Cisplatin 30-min i.v. infusion at 06:00 (morning) and 18:00 (evening)	Total and unbound platin CL 18:00 > 06:00	Leucopenia, neutropenia and nausea symptoms lower at 18:00	[187]
Irinotecan + Oxaliplatin + 5-FU (combined with cetuximab)	Colorectal cancer with liver metastases	Pharmacokinetic study	Hepatic artery infusion (chronomudulated) Irinotecan (180 mg/m^2^)-6 h sinusidal infusion peak at 05:00 h, Oxaliplatin (85 mg/m^2^)-12 h sinusoidal infusion peak at 16:00, 5-FU (2800 mg/m^2^)-12 h sinusoidal infusion peak at 04:00 h	Good correlation between AUC of irinotecan, SN-38, ultrafiltrated Pt and leukopenia. The AUC and C_max_ of ultrafiltrated Pt were significantly correlated with the severity of diarrhea	-	[183]
5-FU, LV and oxaliplatin	Colon or rectum cancers with metastases	Meta-analysis of three Phase III trials	ChronoFLO = 5-FU-LV from 2215 to 09:45 h with a peak at 04:00 h, and oxaliplatin from 10:15 to 21:45 h with a peak at 1600 h.	-	Overall survival was higher in males on chronoFLO when compared with CONV (*p* = 0.009)	[188]
Capecitabine (combined with radiotherapy)	Rectal cancer	Prospective single-center, single-arm Phase II study	Oral Capecitabine (1650 mg/m^2^) 50% dose at 8:00 (morning) and 50% dose at 12:00 (noon)-BRUNCH	-	No Grade 2–3 toxicity of hand-foot syndrome, thrombocytopenia, diarrhea and mucositis. There were no grade IV toxicities	[189]
Capecitabine + Oxaliplatin	Treatment-naïve colorectal cancer patients with metastatic disease	Prospective single-center, single-arm, Phase II study	Oral Capecitabine (2000 mg/m^2^) 50% dose at 8:00 (morning) and 50% dose at 12:00 (noon)-BRUNCH	AUC_0–4 h_ of Capecitabine 08:00 > 12:00 C_max_ of Capecitabine 08:00 > 12:00	Lower incidence of hand-foot syndrome	[190]
Tamoxifen	Breast cancer	Pharmacokinetic cross-over study	Oral 20 or 40 mg once a day at 8:00 or 13:00 or 20:00	AUC_0–8 h_ and C_max_ of tamoxifen 08:00 > 20:00 (20%) tamoxifen t_max_ 08:00 < 20:00 Systemic exposure (AUC0–24 h) to endoxifen 08:00 > 20:00 (15%)	-	[89]
Everolimus (combined with exemastan and tamoxifen)	Metastatic breast cancers	-	Oral morning or evening administration	-	Morning administration of everolimus minimize metabolic alteration and fatigues. No pneumonitis after morning administration	[191]
Sunitinib	Advanced clear cell renal cell carcinoma, Pancreatic neuro-endocrine tumors	Prospective randomized crossover study	8:00. (morning), 13:00 (noon) and 18:00. (evening)	C_trough_ 13:00. or 18:00 p.m. > C_trough_ 8:00 *p* = 0.006	-	[176]
Linifanib	Advanced or metastatic solid tumors and refractory to standard therapy	Phase I, open-label, randomized, crossover study	0.25 mg/kg (maximum 17.5 mg) morning or evening	C_max_ morning > evening (*p* < 0.01)	-	[192]
Radiotherapy	Bone metastases	Cohort study	Treatment times are 08:00–11:00, 11:01–14:00 or 14:01–17:00	-	Females in the 11:01 to 14:00 cohort exhibited higher response rate	[193]

i.v., intravenous; CL, clearance; 5-FU, 5-fluorouracil; LV, leucovorin; CONV, conventional therapy; C_max_, maximum plasma concentration; t_max_, time to reach C_max_; AUC, Area under curve; C_through_, Trough plasma level after reaching steady-state concentration of the drug in repetitive administration; (-), means “not mentioned”.

## Abbreviations

5-FU	5-Fluorouracil
ABC	ATP-Binding Cassette
AGT	O6-alkylguanine transferase
Ahr	Aryl hydrocarbon receptor
ALAS1	Aminolevulinic acid synthase
ASPS	Advanced sleep phase syndrome
ATM	Ataxia telangiectasia mutated
BAX	BCL-2-associated X protein
BMAL1	Brain and muscle aryl hydrocarbon receptor nuclear translocator 1
CAR	Constitutive androstan receptor
CCGs	Clock controlled genes
CDC2	Cyclin-dependent kinase 2
CES	Carboxylesterase
ChronoDDS	Chrono-drug delivery system
ChronoPD	Chronopharmacodynamics
ChronoPK	Chronopharmacokinetics
CKIε	Casein kinase Iε
CLOCK	Circadian locomotor output cycles kaput
Cmax	Maximum plasma concentration
Cry	Cryptochrome
CTS	Circadian timing system
Cyc	Cyclin
CYCLOPS	Cyclic ordering by periodic structure
CYP450	Cytochrome P-450
DBP	Albumin D-site-binding protein
DDS	Drug delivery systems
DPYD	Dihydropyrimidine dehydrogenase
DSPS	Delayed sleep phase syndrome
EGFR	Epidermal growth factor receptor
ERK/MAPK	Extracellular signal-regulated kinase/mitogen-activated protein kinase
FASPS	Familial advanced sleep phase syndrome
GSH	Reduced glutathione
GST	Glutathione-S-transferases
HLF	Hepatic leukemia factor
Hsd3b	3β-hydroxysteroid dehydrogenase
IFN-β	Interferon-β
ISGF	Interferon-stimulated gene factor
MDR1	Multidrug resistance protein 1 (ABCB1, P-glycoprotein, P-gp in human)
Mdr1	Multidrug resistance protein 1 (abcb1a/b mdr1a/b, P-gp in rodents)
MR	Modified Release
Mrp	Multidrug resistance-associated protein (Abcc)
mTOR	Mammalian target of rapamycin
NAT	N-acetyl transferases
NFIL3/E4BP4	Nuclear factor, interleukin 3 regulated
NPAS2	Neuronal PAS-domain protein 2
Oatp	Organic anion transporter polypeptide
Oct	Organic cation transporter
Octn1	Oct novel type 1
p21WAF1	p21 wild-type p53 fragment 1
PARbZip	Proline-acidic amino acid-rich basic leucine zipper
PD	Pharmacodynamics
PDGF	Platelet-derived growth factor
Per	Period
P-gp	P-glycoprotein
PI3K/AKT	Phosphatidylinositol-3-kinase/protein kinase B
PK	Pharmacokinetics
POR	P450 oxidoreductase
PPAR-α	Peroxisome proliferator activated receptor-alpha
PTX	Paclitaxel
PTX-NPs	PTX-loaded polymeric nanoparticles
PXR	Pregnane X receptor
REV-ERB/NR1D	Reverse strand of ERB
RORs	Retinoic acid-receptor-related orphan receptor
SCN	Suprachiasmatic nuclei
SLC	Solute carrier
SULT	Sulfotransferases
TEF	Thyrotroph embryonic factor
Top1	Topoisomerase 1
TS	Thymidylate synthase
UDP	Uridine diphosphate
UGT	Glucuronosyl transferase
VEGF	Vascular endothelial growth factor
WBC	White blood cells
